# Experimental transmission of Zika virus by *Aedes japonicus japonicus* from southwestern Germany

**DOI:** 10.1038/s41426-018-0195-x

**Published:** 2018-11-28

**Authors:** Stephanie Jansen, Anna Heitmann, Renke Lühken, Hanna Jöst, Michelle Helms, Olli Vapalahti, Jonas Schmidt-Chanasit, Egbert Tannich

**Affiliations:** 1 Bernhard Nocht Institute for Tropical Medicine, 20359 Hamburg, Germany; 2 German Centre for Infection Research (DZIF), Partner site Hamburg-Luebeck-Borstel-Riems, 20359 Hamburg, Germany; 3 University of Helsinki and Helsinki University Hospital, 00100 Helsinki, Finland

## Abstract

The invasive mosquito species *Aedes japonicus japonicus* (*Ae. japonicus*) is widely distributed in Central Europe and is a known vector of various arboviruses in the laboratory, including flaviviruses such as Japanese Encephalitis virus or West Nile virus. However, the vector competence of *Ae. japonicus* for the recently emerging Zika virus (ZIKV) has not been determined. Therefore, field-caught *Ae. japonicus* from Germany were orally infected with ZIKV and incubated at 21, 24, or 27 °C to evaluate the vector competence under climate conditions representative of the temperate regions (21 °C) in the species’ main distribution area in Europe and of Mediterranean regions (27 °C). *Aedes japonicus* was susceptible to ZIKV at all temperatures, showing infection rates between 10.0% (21 °C) and 66.7% (27 °C). However, virus transmission was detected exclusively at 27 °C with a transmission rate of 14.3% and a transmission efficiency of 9.5%. Taking into account the present distribution of *Ae. japonicus* in the temperate regions of Central Europe, the risk of ZIKV transmission by the studied *Ae. japonicus* population in Central Europe has to be considered as low. Nevertheless, due to the species’ vector competence for ZIKV and other mosquito-borne viruses, in combination with the possibility of further spread to Mediterranean regions, *Ae. japonicus* must be kept in mind as a potential vector of pathogens inside and outside of Europe.

## Introduction

Zika virus (ZIKV) is an emerging mosquito-borne virus within the family Flaviviridae that was first isolated from sentinel rhesus macaques in Uganda in 1947^1^. After decades of silent circulation, unprecedented ZIKV epidemics occurred in Micronesia, Polynesia, and, finally, in the Americas in 2015; the hundreds of thousands of human cases finally resulted in the announcement of a Public Health Emergency of International Concern through the World Health Organization^2^. Clinical courses associated with ZIKV infections can range from mild clinical symptoms to severe diseases, including neonatal microcephaly and neurological disorders such as Guillain-Barré syndrome^3^. The mosquito species *Aedes aegypti* and *Aedes albopictus* are considered the primary and secondary vectors of ZIKV; however, a wide variety of other *Aedes* species have been identified as potentially susceptible to ZIKV infection^4^. Recent experimental studies suggested that only *Ae. albopictus* might play a role in ZIKV transmission in Central Europe, while common members of the genus *Culex* are probably not important^5,6^. However, north of the Alps, the Asian tiger mosquito is currently established at only a few sites, with relatively low abundance^7,8^. By contrast, the invasive Asian bush mosquito *Aedes japonicus japonicus* (*Ae. japonicus*) is widely distributed in Central Europe and is currently established in at least 10 countries, including large parts of Germany^8^. In 2008, the first invasive spreading of *Ae. japonicus* in Europe was reported in Switzerland^9^, and *Ae. japonicus* is now listed as one of the nine most dominant mosquito species in Switzerland. Shortly after its introduction in Switzerland, *Ae. japonicus* was first reported in Germany, followed by the establishment of populations at several sites. In the Netherlands and Belgium, mosquito control programs have been initiated due to the massive *Ae. japonicus* populations^9–12^.

*Ae. japonicus* is a container-dwelling species, colonizing both natural (e.g., bamboo stubs and tree holes) and man-made (e.g., tires and barrels) breeding sites^13^. Due to its tolerance of rather low temperatures, *Ae. japonicus* has a relatively long seasonal activity compared to other container-breeders^14^. *Ae. japonicus* has an opportunistic feeding pattern with a preference for mammals, including humans, although avian host species have also been reported^15,16^. Thus, *Ae. japonicus* could potentially serve as a bridge vector for zoonotic arboviruses. The species is an experimentally proven vector of several flaviviruses, including Japanese Encephalitis virus (JEV), West Nile virus (WNV), and Saint Louis encephalitis virus^17–19^, as well as arboviruses of other families, such as La Crosse virus (LACV, *Peribunyaviridae*) and Chikungunya virus (CHIKV, *Togaviridae*)^20,21^. Previous studies with an *Ae. japonicus* population from southwestern Germany also revealed a vector competence for JEV under laboratory conditions^19^.

In light of the continuing spread of *Ae. japonicus* in Europe and the ongoing circulation of ZIKV in America, the aim of this study was to evaluate whether *Ae. japonicus* has vector competence for ZIKV under climate conditions representative of tropical and temperate regions.

## Results

To assess the suitability of the collected *Ae. japonicus* for vector competence studies, a small number of specimens were challenged with JEV in a preliminary study. In agreement with previous findings^19^, the *Ae. japonicus* specimens from southwestern Germany were susceptible to JEV. The infection rate (IR) was 51.9%, with an average amount of viral RNA of 5.6 × 10^8^ copies/specimen (*n* = 27). In addition to the previous experiment, we also investigated the transmission of infectious virus particles by analyzing mosquito saliva following incubation of infected mosquitoes at 27 °C for 14 days. The results indicated a transmission rate (TR) of 78.6%.

**Fig. 1:**
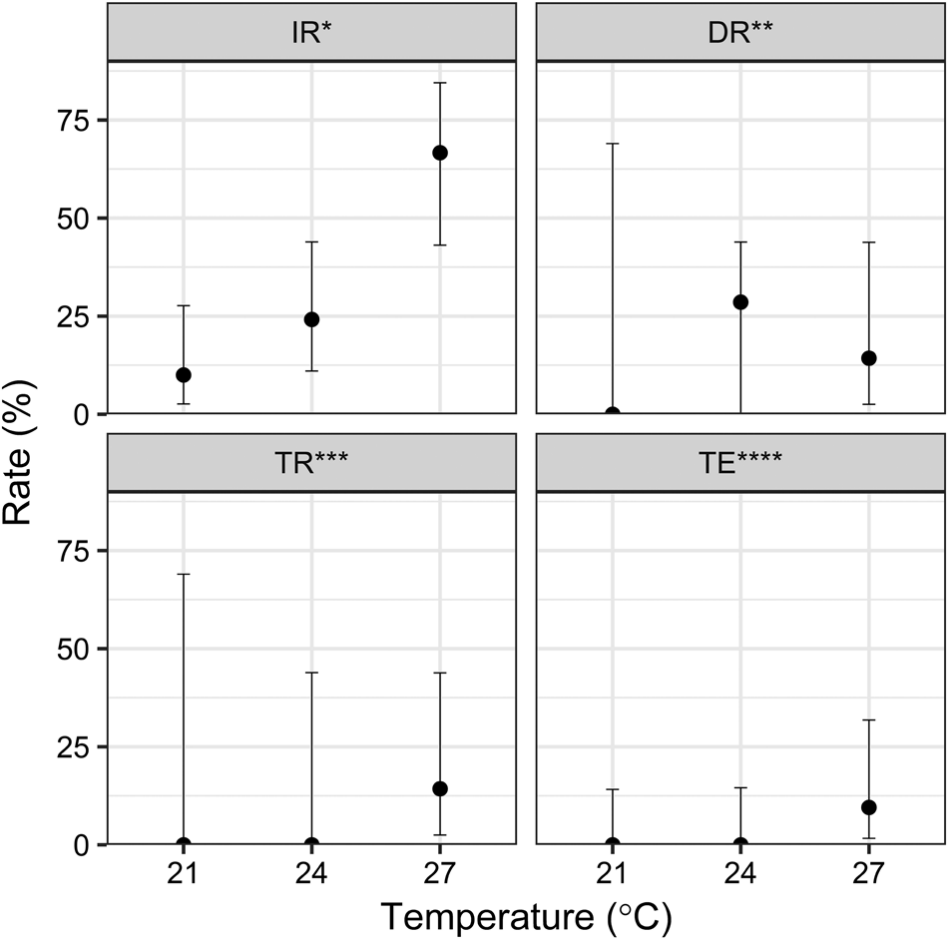
Means and 95% confidence intervals for the ZIKV infection, dissemination, and transmission rates as well as the transmission efficiency of *Ae. japonicus* from southwestern Germany as revealed by analyses of bodies (IR), legs (DR), and saliva (TR and TE) following challenge of mosquitoes with infectious blood meals and incubation at 21 °C (*n* = 30), 24 °C (*n* = 29), or 27 °C (*n* = 21) for 2 weeks. Three independent trials were performed for each temperature. *Infection rate (IR): number of ZIKV-positive mosquito bodies per number of fed females. **Dissemination rate (DR): number of mosquitoes with ZIKV-positive legs per number of ZIKV-positive mosquito bodies. ***Transmission rate (TR): number of mosquitoes with ZIKV-positive saliva per number of ZIKV-positive mosquito bodies. ****Transmission efficiency (TE): number of mosquitoes with ZIKV-positive saliva per total number of fed females

Subsequently, mosquitoes were analyzed for ZIKV infection. Fourteen days post infection, ZIKV RNA was present in the bodies of challenged *Ae. japonicus* at all of the tested temperatures (Fig. 1). The relative numbers of ZIKV-positive mosquitoes (Fig. 1) and the amount of viral RNA (Table 1) increased with increasing incubation temperatures. The IR increased from 10% (3/30) at 21 °C to 24.1% (7/29) at 24 °C and to 66.7% (14/21) at 27 °C. This pattern is also reflected in the amount of virus RNA within the mosquito bodies, which increased from 1.2 × 10^4^ RNA copies/specimen at 21 °C to 2.6 × 10^6^ RNA copies/specimen at 24 °C to 6.4 × 10^8^ RNA copies/specimen at 27 °C (Table 1). Dissemination of the virus was found in mosquitoes kept at 24 and 27 °C but not in mosquitoes incubated at 21 °C. However, the averaged leg titers were substantially higher at 27 °C (6.4 × 10^8^ RNA copies/specimen) than at 24 °C (8.4 × 10^2^ RNA copies/specimen) (Table 1). This is also reflected by the detection of infectious virus particles in the saliva of two mosquitoes kept at 27 °C, resulting in a TR of 14.3% (2/14) and a transmission efficiency of 9.5% (2/21).

**Table 1:** Calculation of the virus titers from bodies or legs for *Ae. japonicus* specimens from southwestern Germany following challenge of mosquitoes with infectious blood meals and incubation at 21 , 24, or 27 °C for 2 weeks

Temperature in °C	Body titer, mean (SD) log10 RNA copies/specimen	Leg titer, mean (SD) log10 RNA copies/specimen
21	4.6 (1.0)	0.0 (0.0)
24	4.9 (1.5)	2.9 (2.9)
27	5.9 (1.8)	4.2 (4.2)

## Discussion

To the best of our knowledge, this is the first study on the vector competence of *Ae. japonicus* for ZIKV (see review by Epelboin et al.^4^), and our results show a low transmission efficiency at high temperatures. The species is a known competent vector of a variety of arboviruses, including flaviviruses (e.g., WNV, JEV, or Dengue virus), as well as members of other virus families, including *Peribunyaviridae* (LACV) and *Togaviridae* (CHIKV)^17,19–21^.

Over the last two decades, *Ae. japonicus* has successfully invaded Central European countries as well as large parts of North America, and it is found primarily in areas with predominantly temperate climate conditions^8,13,14,22,23^. *Ae. japonicus* eggs are resistant to frost and desiccation. Furthermore, the seasonal activity of the species is longer than that of other container-breeding species. Due to these attributes, *Ae. japonicus* has some developmental advantages over native species that could affect mosquito population patterns as well as pathogen transmission in newly colonized regions. Likewise, it is of considerable interest to collect information on the vector competence of *Ae. japonicus* for newly emerging viruses such as ZIKV, with a special emphasis on the temperate climate conditions of the species’ current distribution range.

Previous studies described relatively low feeding rates of field-caught *Ae. japonicus* using saturated cotton sticks or feeding systems with chicken skin^19,21^. The experiments presented here demonstrate that artificial feeding via blood drops seems to be an efficient alternative for field-caught *Ae. japonicus* mosquitoes, resulting in a feeding rate of 75%. The experimental results clearly indicate temperature-dependent variations in the susceptibility of *Ae. japonicus* to ZIKV. Following the experimental challenge with ZIKV-containing blood meals, the number of infected specimens as well as the amount of ZIKV RNA copies per mosquito increased with increasing incubation temperatures (21 °C < 24 °C < 27 °C). This result is consistent with our previous studies performed with ZIKV, where the IRs of various mosquito species from Central Europe were also temperature-dependent^6^. Dissemination was only observed at 24  and 27 °C. Infectious virus particles were exclusively detected in two mosquito specimens incubated at an elevated temperature of 27 °C, resulting in a TR of 14.3%. There is a high variability in the range of TRs at tropical incubation temperatures (26–28 °C) for both *Ae. aegypti* (21–87%) and *Ae. albopictus* (18–77%)^6,24–28^. Nevertheless, both species showed considerably higher TRs than *Ae. japonicus*; whereas *Ae. vexans* also has a low vector competence for ZIKV, with a TR between 2 and 7%^29^. Likewise, the transmission efficiency of 9.5% for *Ae. japonicus* at the tropical temperature is lower than the known transmission efficiency for *Ae. aegypti* (26%) under similar conditions^27^. Previous vector competence studies with *Ae. japonicus* and various arboviruses were only performed under tropical temperature conditions (i.e., 25–28 °C)^17–21^. Therefore, it is unknown if the lack of virus transmission at lower temperatures is a general feature for viral transmission by *Ae. japonicus* or if this observation is specific for ZIKV. By contrast, the primary and secondary vectors *Ae. aegypti* and *Ae. albopictus* can transmit ZIKV below 27 °C. Viable ZIKV virus particles were detected in the saliva of *Ae. aegypti* even at 20 °C^30^. Therefore, the lack of transmission by *Ae. japonicus* is a species-specific observation and not a general pattern for ZIKV.

One explanation for the lack of ZIKV transmission by *Ae. japonicus* at temperatures below 27 °C might be a combination of higher virus replication rates and species-specific, temperature-dependent effects on the mosquito microbiome or immune regulatory pathways^31^. However, as shown before, vector competence is influenced by a three-way interaction between the vector population, the virus strain, and temperature^32,33^. Only a suitable combination of these factors allows the virus to replicate and disseminate to the salivary glands to enable transmission through the next bite. However, studies with a combination of one specific mosquito population with one specific virus strain must be interpreted with caution. Even studies with the same combination of vector species and virus can come to varying TRs or even to inconsistent results regarding the species’ susceptibility to a virus. Studies performed with either field-caught *Ae. aegypti* or *Ae. albopictus* populations from different sites within one country revealed clearly varying TRs for the same ZIKV strains^34,35^. In addition, the vector competence of the same mosquito population can be highly strain-specific. *Ae. aegypti* from Mexico showed an increased vector competence for African strains of ZIKV compared with an American ZIKV strain^36,37^. Studies with the WNV strain NY99 and *Ae. japonicus* mosquitoes from northern Switzerland revealed transmission of WNV^38^. By contrast, *Ae. japonicus* mosquitoes from southwestern Germany were shown to be refractory to the same WNV strain^19^. Similar contradictions have been discussed regarding the vector competence of *Culex quinquefasciatus* for ZIKV, where some studies detected transmission while others did not^39–41^. These differences might be explained by variations in the experimental setup, e.g., the origin of the mosquito population, the virus strain, or vector maintenance protocols in the laboratory^42^. Thus, standardized studies to investigate the vector competence for different local mosquito population/virus strain combinations must be considered to allow a thorough risk assessment. In particular, further analysis of the *Ae. japonicus* populations from northern America/Asia should be performed to assess the risk of ZIKV transmission in these regions.

Currently, the distribution of *Ae. japonicus* in Europe is primarily restricted to regions with temperate climates^13^. The lack of vector competence at temperatures below 27 °C suggests a limited risk for ZIKV transmission by *Ae. japonicus* in Europe. However, the rapid spread of *Ae. japonicus* in Northeast America (as far as 30°N′ latitude in Florida) and the native distribution in Asia at the same latitude illustrate the risk for the species to spread to the Mediterranean region^14,43^. *Ae. japonicus* may adapt to new environmental conditions and might also have the potential to invade areas of higher temperatures in the Mediterranean region, as has already happened in North America^13^. Nevertheless, for a comprehensive risk assessment of ZIKV transmission in Central Europe, *Aedes* species such as the native mosquito species *Aedes vexans* and the invasive species *Aedes koreicus* should also be considered and investigated as potential ZIKV vectors under temperate climate conditions. *Ae. vexans* from North America was proven to have a transmission potential for ZIKV of approximately 1–5% at incubation temperatures of 28 or 27 °C^29,44^. These low TRs must be considered in light of the locally very high mosquito abundance and aggressive human-biting behavior along rivers in Central Europe^45^. Therefore, further studies should investigate whether this species can transmit ZIKV at lower temperatures. Another candidate of interest is the invasive mosquito species *Ae. koreicus*, which is closely related to *Ae. japonicus*. *Ae. koreicus* is also a vector for arboviruses such as JEV^46^ or CHIKV^23^ and was quite recently introduced into Central Europe, including in Germany^47–49^.

In conclusion, transmission of ZIKV by *Ae. japonicus* appears to be limited to elevated temperatures. Nevertheless, due to the demonstrated species’ vector competence for ZIKV and for other mosquito-borne viruses, in combination with a possible further spread to southern Europe, *Ae. japonicus* must be considered a potential vector of pathogens, including ZIKV.

## Materials and methods

### Source, rearing, and experimental infection of mosquitoes

*Ae. japonicus* eggs were collected with ovitraps in southwestern Germany (49°31′26.26″N, 8°40′16.88″E) in summer 2017. Approximately 1200 eggs were flooded in the laboratory, and the larvae and adults were maintained at 26 °C, with a relative humidity of 80% and a 12:12 light:dark photoperiod. These temperature conditions were selected because the larval development of *Ae. japonicus* is known to positively correlate with increasing temperatures up to 30 °C. As the pupation limit is reached at 28 °C, we chose 26 °C as the incubation temperature for successful and rapid development of the mosquitoes^14,50^. Species identification was performed using the morphological key in the “Guidelines for the surveillance of invasive mosquitoes in Europe”^51^. To exclude natural flavivirus infections that could potentially interfere with the experimental outcome, 10 randomly selected adult specimens were tested with pan-Flavi-, pan-Bunya-, and pan-Alphavirus PCRs, but these tests were negative^52–54^.

Groups of 20 females (4–14 days old) were placed in plastic vials, starved for 24 h, and challenged with infectious blood meals. The feeding, incubation, and analysis of the mosquitoes were performed in the BSL-3 insectary in the Bernhard Nocht Institute for Tropical Medicine, Hamburg, Germany. To support a high feeding rate, we provided infectious blood in 50 µl drops at the bottoms of the vials (two drops per vial). Thus, a feeding rate of 75% (i.e., the percentage of engorged females to total females) was reached.

For validation of the salivation assay for *Ae. japonicus*, an infection experiment with JEV was first performed. Female mosquitoes were infected via an infectious blood meal using the SA-14 strain of JEV (GenBank accession number EU073992)^55^ at a final concentration of 10^7^ plaque-forming units/milliliter (PFU/ml) and were kept at 27 °C for 14 days.

Subsequently, a total of 381 female mosquitoes were challenged with blood meals containing ZIKV, strain ZIKV_FB-GWUH-2016 (GenBank accession number KU870645, fifth passage)^56^ at a final concentration of 10^7^ PFU/ml. Two hundred forty-three engorged females were incubated at 80% humidity and temperatures of 21, 24, or 27 °C.

### Assessment of ZIKV infection, dissemination, and transmission

Fourteen days post infection, mosquitoes were analyzed for JEV (*n* = 27) or ZIKV (*n* = 79) infection, dissemination, and transmission. Infection, dissemination, and virus titers were determined by separate analyses of mosquito bodies and heads without legs and wings (infection and body titer) and of legs (dissemination and leg titer) for the presence of viral ZIKV RNA using a quantitative real-time PCR assay (qRT-PCR; Real Star Zika Virus RT-PCR Kit, Altona Diagnostics, Hamburg, Germany). ZIKV transmission was assessed by testing mosquito saliva for the presence of infectious virus particles using the salivation assay as previously described^6^. In short, mosquitoes were immobilized and the probosces were placed into a filter tip containing 10 µl of phosphate-buffered saline (PBS). After 30 min, saliva-containing PBS was pipetted into the media of Vero cells seeded in a 96-well plate to measure the cytopathic effect, i.e., the presence of infectious virus particles, after 7 days. The presence of ZIKV in the supernatant of cytopathic cells was subsequently tested by the abovementioned qRT-PCR assay.

### Statistical analysis

Calculations of the IR, dissemination rate (DR), and TR were performed as described by Fortuna et al.^57^. The IR is defined as the number of virus-positive mosquito bodies per number of fed females, the DR is defined as the number of virus-positive legs per number of virus-positive bodies, and the TR is defined as the number of virus-positive saliva samples per number of virus-positive bodies. Calculation of the transmission efficiency was conducted as described by Chouin-Carneiro et al.^35^ and is defined as the number of virus-positive saliva samples per total number of fed females. The R program^58^ was used for all calculations and visualizations, including the *ggplot2*^59^, *tidyr*^60^, and *plyr*^61^ packages.

## Data Availability

All relevant data are provided within the paper.

## References

[1] Dick GWA, Kitchen SF, Haddow AJ (1952). Zika virus. I. Isolations and serological specificity. Trans. R. Soc. Trop. Med. Hyg..

[2] WHO. WHO Director-General summarizes the outcome of the Emergency Committee regarding clusters of microcephaly and Guillain-Barré syndrome. At <http://www.who.int/en/news-room/detail/01-02-2016-who-director-general-summarizes-the-outcome-of-the-emergency-committee-regarding-clusters-of-microcephaly-and-guillain-barr%c3%a9-syndrome> (2016).

[3] Musso D, Gubler DJ (2016). Zika virus. Clin. Microbiol. Rev..

[4] Epelboin Y, Talaga S, Epelboin L, Dusfour I (2017). Zika virus: an updated review of competent or naturally infected mosquitoes. PLoS Negl. Trop. Dis..

[5] Boccolini D (2016). Experimental investigation of the susceptibility of Italian *Culex pipiens* mosquitoes to Zika virus infection. Eur. Surveill..

[6] Heitmann A (2017). Experimental transmission of Zika virus by mosquitoes from central Europe. Eur. Surveill..

[7] Becker N (2017). First mass development of *Aedes albopictus* (Diptera: Culicidae)-its surveillance and control in Germany. Parasitol. Res..

[8] European Centre for Disease Prevention and Control (ECDC), European Food Safety Authority (EFSA). VectorNet: a European network for sharing data on the geographic distribution of arthropod vectors, transmitting human and animal disease agents. At <https://ecdc.europa.eu/en/about-us/partnerships-and-networks/disease-and-laboratory-networks/vector-net> (accessed November 10, 2018).

[9] Schaffner F, Kaufmann C, Hegglin D, Mathis A (2009). The invasive mosquito *Aedes japonicus* in Central Europe. Med. Vet. Entomol..

[10] European Centre for Disease Prevention and Control. *Aedes japonicus*—factsheet for experts. https://ecdc.europa.eu/en/disease-vectors/facts/mosquito-factsheets/aedes-japonicus (2018).

[11] Ibanez-Justicia A (2018). The effectiveness of Asian bush mosquito (*Aedes japonicus japonicus*) control actions in colonised peri-urban areas in the Netherlands. J. Med. Entomol..

[12] Wagner S, Guidi V, Torgerson PR, Mathis A, Schaffner F (2018). Diversity and seasonal abundances of mosquitoes at potential arboviral transmission sites in two different climate zones in Switzerland. Med. Vet. Entomol..

[13] Medlock JM (2015). An entomological review of invasive mosquitoes in Europe. Bull. Entomol. Res..

[14] Kaufman MG, Fonseca DM (2014). Invasion biology of *Aedes japonicus japonicus* (*Diptera*: *Culicidae*). Annu. Rev. Entomol..

[15] Molaei G, Farajollahi A, Scott JJ, Gaugler R, Andreadis TG (2009). Human bloodfeeding by the recently introduced mosquito, *Aedes japonicus japonicus*, and public health implications. J. Am. Mosq. Control Assoc..

[16] Schönenberger AC (2016). Host preferences in host-seeking and blood-fed mosquitoes in Switzerland. Med. Vet. Entomol..

[17] Turell MJ, O’Guinn ML, Dohm DJ, Jones JW (2001). Vector competence of North American mosquitoes (Diptera: Culicidae) for West Nile virus. J. Med. Entomol..

[18] Sardelis MR, Turell MJ, Andre RG (2003). Experimental transmission of St. Louis encephalitis virus by *Ochlerotatus j. japonicus*. J. Am. Mosq. Control Assoc..

[19] Huber K (2014). *Aedes japonicus japonicus* (Diptera: Culicidae) from Germany have vector competence for Japan encephalitis virus but are refractory to infection with West Nile virus. Parasitol. Res..

[20] Sardelis MR, Turell MJ, Andre RG (2002). Laboratory transmission of La Crosse virus by *Ochlerotatus j. japonicus* (Diptera: Culicidae). J. Med. Entomol..

[21] Schaffner F, Vazeille M, Kaufmann C, Failloux AB, Mathis A (2011). Vector competence of *Aedes japonicus* for chikungunya and dengue viruses. Eur. Mosq. Bull..

[22] Andreadis TG, Wolfe RJ (2010). Evidence for reduction of native mosquitoes with increased expansion of invasive *Ochlerotatus japonicus japonicus* (Diptera: Culicidae) in the northeastern United States. J. Med. Entomol..

[23] Ciocchetta S (2018). The new European invader Aedes (Finlaya) koreicus: a potential vector of chikungunya virus. Pathog. Glob. Health.

[24] Richard V, Paoaafaite T, Cao-Lormeau VM (2016). Vector competence of French Polynesian *Aedes aegypti* and *Aedes polynesiensis* for ZikaVirus. PLoS Negl. Trop. Dis..

[25] Goertz GP, Vogels CBF, Geertsema C, Koenraadt CJM, Pijlman GP (2017). Mosquito co-infection with Zika and chikungunya virus allows simultaneous transmission without affecting vector competence of A*edes aegypti*. PLoS Negl. Trop. Dis..

[26] Duchemin JB (2017). Zika vector transmission risk in temperate Australia: a vector competence study. Virol. J. Engl..

[27] Di Luca, M. et al. Experimental studies of susceptibility of Italian *Aedes albopictus* to Zika virus. *Euro Surveill.* **21**, ES.2016.21.18.30223 (2016). 10.2807/1560-7917.ES.2016.21.18.30223 27171034

[28] Hall-Mendelin S (2016). Assessment of local mosquito species incriminates *Aedes aegypti* as the potential vector of Zika virus in Australia. PLoS Negl. Trop. Dis..

[29] Gendernalik A (2017). American *Aedes vexans* mosquitoes are competent vectors of Zika virus. Am. J. Trop. Med. Hyg..

[30] Tesla, B. et al. Temperature drives Zika virus transmission: evidence from empirical and mathematical models. *bioRxiv*. <http://biorxiv.org/content/early/2018/04/27/259531.abstract> (2018). 10.1098/rspb.2018.0795 PMC6111177 30111605

[31] Murdock CC, Paaijmans KP, Cox-Foster D, Read AF, Thomas MB (2012). Rethinking vector immunology: the role of environmental temperature in shaping resistance. Nat. Rev. Microbiol..

[32] Tabachnick WJ (2013). Nature, nurture and evolution of intra-species variation in mosquito arbovirus transmission competence. Int. J. Environ. Res. Public Health.

[33] Zouache K (2014). Three-way interactions between mosquito population, viral strain and temperature underlying chikungunya virus transmission potential. Proc. R. Soc. B.

[34] Garcia-Luna SM (2018). Variation in competence for ZIKV transmission by *Aedes aegypti* and *Aedes albopictus* in Mexico. PLoS Negl. Trop. Dis..

[35] Chouin-Carneiro T (2016). Differential susceptibilities of *Aedes aegypti* and *Aedes albopictus* from the Americas to Zika virus. PLoS Negl. Trop. Dis..

[36] Willard AK (2017). Zika virus exhibits lineage-specific phenotypes in cell culture, in *Aedes aegypti* mosquitoes, and in an embryo model. Viruses.

[37] Weger-Lucarelli J (2016). Vector competence of American mosquitoes for three strains of Zika virus. PLoS Negl. Trop. Dis..

[38] Veronesi E (2018). Experimental evaluation of infection, dissemination, and transmission rates for two West Nile virus strains in European *Aedes japonicus* under a fluctuating temperature regime. Parasitol. Res..

[39] Guo XX (2016). *Culex pipiens quinquefasciatus*: a potential vector to transmit Zika virus. Emerg. Microbes Infect..

[40] Roundy CM (2017). Lack of evidence for Zika virus transmission by *Culex* mosquitoes. Emerg. Microbes Infect..

[41] Ayres C (2017). Response to: ‘Lack of evidence for Zika virus transmission by *Culex* mosquitoes’. Emerg. Microbes Infect..

[42] Wilson AJ, Harrup LE (2018). Reproducibility and relevance in insect-arbovirus infection studies. Curr. Opin. Insect Sci..

[43] Riles MT (2017). First record of *Aedes japonicus* in Florida. J. Am. Mosq. Control Assoc..

[44] O’Donnell KL, Bixby MA, Morin KJ, Bradley DS, Vaughan JA (2017). Potential of a northern population of *Aedes vexans* (Diptera: Culicidae) to transmit Zika Virus. J. Med. Entomol..

[45] Becker, N. et al. *Mosquitoes and Their Control* (Springer, Berlin Heidelberg, 2010).

[46] Miles JA (1964). Some ecological aspects of the problem of arthropod-borne animal viruses in the Western Pacific and South-East Asia regions. Bull. World Health Organ..

[47] Montarsi F (2013). Distribution and habitat characterization of the recently introduced invasive mosquito *Aedes koreicus* [*Hulecoeteomyia koreica*], a new potential vector and pest in north-eastern Italy. Parasit. Vectors.

[48] Versteirt V (2012). Bionomics of the established exotic mosquito species *Aedes koreicus* in Belgium, Europe. J. Med. Entomol..

[49] Werner D, Zielke DE, Kampen H (2016). First record of *Aedes koreicus* (Diptera: Culicidae) in Germany. Parasitol. Res..

[50] Scott, J. J. *The Ecology of the Exotic Mosquito Ochlerotatus (Finlaya) japonicus japonicus (Theobald1901) (Diptera: Culicidae) and an Examination of its Role in the West Nile Virus cycle in New Jersey.* Ph.D. thesis. Rutgers Univ. (2003).

[51] European Centre for Disease Prevention and Control (ECDC). Guidelines for the surveillance of invasive mosquitoes in Europe. <https://ecdc.europa.eu/sites/portal/files/media/en/publications/Publications/TER-Mosquito-surveillance-guidelines.pdf> (2012). 22971331

[52] Chao DY, Davis BS, Chang GJJ (2007). Development of multiplex real-time reverse transcriptase PCR assays for detecting eight medically important flaviviruses in mosquitoes. J. Clin. Microbiol..

[53] Eshoo MW (2007). Direct broad-range detection of alphaviruses in mosquito extracts. Virology.

[54] Lambert AJ, Lanciotti RS (2009). Consensus amplification and novel multiplex sequencing method for S segment species identification of 47 viruses of the *Orthobunyavirus*, *Phlebovirus*, and *Nairovirus* genera of the family *Bunyaviridae*. J. Clin. Microbiol..

[55] Moureau G (2007). A real-time RT-PCR method for the universal detection and identification of flaviviruses. Vector Borne Zoonotic Dis..

[56] Driggers RW (2016). Zika virus infection with prolonged maternal viremia and fetal brain abnormalities. N. Engl. J. Med..

[57] Fortuna C (2015). Experimental studies on comparison of the vector competence of four Italian *Culex pipiens* populations for West Nile virus. Parasit. Vectors.

[58] R Core Team. R: a language and environment for statistical computing. <http://www.r-project.org/_> (2014).

[59] Wickham, H. *ggplots2: Elegant Graphics for Data Analysis* (Springer International Publishing, New York, 2016).

[60] Wickham, H. & Henry, L. tidyr: easily tidy data with ‘spread()’ and ‘gather()’ functions. At <https://cran.r-project.org/web/packages/tidyr/> (2017).

[61] Wickham H (2011). The split-apply-combine strategy for data analysis. J. Stat. Softw..

